# Frequency and Predictors of Virtual Visits in Patients With Heart Failure Within a Large Health System: Retrospective Cohort Study

**DOI:** 10.2196/70414

**Published:** 2025-08-12

**Authors:** Anna M Maw, Garth C Wright, Meagan R Bean, Larry A Allen, Daniel D Matlock, Lilia Cervantes, Russell E Glasgow, Amy G Huebschmann, Kathryn L Colborn, Thomas K Houston, Katie E Trinkley

**Affiliations:** 1 Division of Hospital Medicine, University of Colorado Anschutz Medical Campus, 12605 E 16th Ave, Aurora, CO, 80045, United States, 1 917-246-0702; 2 Adult and Child Center for Outcomes Research and Delivery Science Center, University of Colorado Anschutz Medical Campus, Aurora, CO, United States; 3 Skaggs School of Pharmacy and Pharmaceutical Sciences, University of Colorado Anschutz Medical Campus, Aurora, CO, United States; 4 Department of Medicine, School of Medicine, University of Colorado Anschutz Medical Campus, Aurora, CO, United States; 5 VA Eastern Colorado Geriatric Research Education and Clinical Center, Denver, CO, United States; 6 Department of Family Medicine, School of Medicine, University of Colorado Anschutz Medical Campus, Aurora, CO, United States; 7 Department of Biostatistics and Informatics, Colorado School of Public Health, Aurora, United States; 8 School of Medicine, Wake Forest University, Winston-Salem, United States; 9 Atrium Health Wake Forest Baptist Comprehensive Cancer Center, Winston-Salem, NC, United States; 10 Department of Biomedical Informatics, School of Medicine, University of Colorado Anschutz Medical Campus, Aurora, United States; 11 Colorado Center for Personalized Medicine, School of Medicine, University of Colorado Anschutz Medical Campus, Aurora, United States

**Keywords:** telehealth, virtual care, health equity, heart failure, digital technology

## Abstract

**Background:**

Virtual care interventions have the potential to improve access to care and serial medication intensification for patients with chronic heart failure with reduced ejection fraction (HFrEF). However, concerns remain that these interventions might unintentionally create or widen existing disparities in care delivery and patient outcomes.

**Objective:**

This study aimed to characterize the health care use patterns of patients who have HFrEF, including specialty type and frequency of in-person and virtual visits.

**Methods:**

We conducted a retrospective cohort study of patients with HFrEF within a large health system. Inclusion criteria were patients alive with an ejection fraction ≤40% as of September 1, 2021, and at least one virtual or in-person outpatient visit to a primary care or cardiology clinician in the prior year. Descriptive statistics were used to evaluate baseline patient demographics and clinical use data and outcomes. Univariate analyses were performed both with virtual visits as a variable (received or did not receive) using the chi-square test for association and as a discrete outcome using the Wilcoxon rank-sum test to capture potentially important predictor variables that could influence use or frequency of using virtual visits. The primary outcome of interest was the odds of at least one virtual visit during the 1-year evaluation period from 2021 to 2022. Descriptive statistics were used to evaluate baseline patient demographics and care use. A logistic regression model was used to model at least one primary care or cardiology virtual visit.

**Results:**

A total of 8481 patients were included in the analysis. The mean age was 65.9 years (SD 15.1), 5672 (66.9%) patients were male and 6608 (77.9%) patients were non-Hispanic White. The majority of patients had no cardiology (7938/8481, 93.6%) or primary care (7955/8481, 93.8%) virtual visits during the evaluation period. Multivariable logistic regression showed significantly higher odds of having at least one virtual visit for patients with certain digital access—for example, email on file (odds ratio [OR] 9.3, *P*≤.001), cell phone on file (OR 2.9, *P*≤.001), and active electronic health record patient portal (OR 2.8, *P*≤.001)—than those without. Age, race, ethnicity, rurality, and Social Vulnerability Index were not associated with virtual visits.

**Conclusions:**

Only a minority of patients with HFrEF were seen via virtual visits. Patients who regularly used digital technology were more likely to have virtual visits. Patients were more likely to be seen in a cardiology clinic than by a primary care provider. Although there was no evidence of an association between social determinants of health factors like race, ethnicity, or rurality with digital divide indicators, these findings should be interpreted with caution given the limitations of these data. Future studies should aim to replicate the findings of this study and explore ways to enhance the effective and equitable use of virtual visits.

## Introduction

Virtual care interventions have been identified as tools with the potential to enhance access to care and increase guideline-directed medical therapy prescribing for patients with heart failure with reduced ejection fraction (HFrEF) [1,2]. Patients with HFrEF require frequent visits with their clinicians in order to monitor volume status and titrate their medications. Virtual visits have the potential to reduce the burden of transportation and facilitate timely evaluation by a clinician. However, it is unclear the extent to which patients with HFrEF access virtual care and if there are differences in virtual care use based on patient characteristics. There are concerns that the use of virtual health interventions could create or widen existing disparities in care delivery and patient outcomes, notably for populations who have historically been underrepresented [3].

One potential disparity is related to the “digital divide,” which refers to gaps in both access to and use of digital technologies that can lead to disparities in health outcomes [4]. Some determinants of the digital divide include electronic health record (EHR) patient portal inactivity and not having an email or phone number on file within the EHR. These gaps have been attributed to multiple dynamic factors such as lack of access to internet and digital devices as well as low digital literacy—knowledge and skill in the use of digital tools [5]. Older adults are particularly at risk for digital exclusion due to potentially lower digital literacy, which in turn can lead to reluctance to engage with some health care services [6,7]. This is a particularly important consideration in heart failure given that heart failure is more common among older adults [8]. Understanding patient characteristics associated with the digital divide in heart failure care is crucial for developing targeted interventions to bridge these gaps and ensure equitable access to care for all individuals affected by heart failure, particularly those patients living in rural areas or those for whom driving or access to transportation is a challenge.

Perhaps initially driven by the COVID pandemic, the use of telehealth continues to grow on a global level, and it is likely that as technology evolves, the integration of telehealth specifically into the treatment and management of patients with HFrEF will increasingly be used to enhance patient care and lessen the burden of frequent visits. As health care transitions to a hybrid model combining both in-person and virtual visits, there is a need to evaluate how patients with HFrEF receive care and assess the equity of care provided across different patient populations and with consideration of the digital divide. One health system reported an increase in telehealth visits per month from less than 20 in 2016 to over 70,000 across all 12 hospitals within the health system in 2023 [9]. Although it is known that during the height of the pandemic, almost all outpatient care for patients with HFrEF was virtual [10], a question not yet addressed in the literature is the extent to which outpatient care for patients with HFrEF remains virtual and if there are disparities in who receives virtual care that need to be addressed or further evaluated to ensure equitable care. In response to this gap in the literature, this study aims to assess how care is received by patients with HFrEF postpandemic, including the extent to which patients continue to use virtual visits and if there are differences in virtual visits or subspecialty use by patient characteristics such as race, ethnicity, rurality, or digital divide measures.

## Methods

### Study Design and Setting

This was a retrospective cohort study conducted at the UCHealth health system, which is a large regional health system in Colorado and areas of Wyoming and Nebraska. UCHealth is comprised of 14 hospitals, 900 clinics, and over 6000 physicians who serve academic, rural, suburban, and community settings. The entire system is serviced by a single instance of the Epic EHR software program (Epic Systems, Verona, WI, United States).

### Ethical Considerations

This protocol, #22-1154, was approved by the University of Colorado Multiple Institutional Review Board. This was a secondary analysis of deidentified patient data.

### Data Source

We collected data on eligible patients from Health Data Compass, our institutional enterprise data warehouse that extracts, integrates, and delivers data from the EHR for patients within the UCHealth system. Data from Health Data Compass also includes data from the public health department for Colorado and US census data, which were used to supplement EHR data related to death dates and location of residence (ie, rural, urban). The primary outcome of interest was the odds of at least one virtual visit, defined as a synchronous meeting between a clinician and patient, during the 1-year evaluation period from September 1, 2021, to August 31, 2022.

### Patient Population

Patients meeting the following criteria were included in our study: alive as of September 1, 2021 with a history of an ejection fraction (EF) ≤40%, and at least one virtual or in-person outpatient visit to a primary care or cardiology clinician in the UCHealth system in the prior year (between September 1, 2020 and August 30, 2021).

### Visit Type

Cardiology and primary care clinicians were defined based on department specialty. Among these specialties, outpatient visit types were limited to those that were used by physicians and advanced practice clinicians (eg, excluded nurse or lab-only visit types).

### Outcomes

The primary outcome of interest was the odds of at least one virtual visit versus no virtual visit during the 1-year evaluation period from September 1, 2021, through August 31, 2022. This period was chosen to reflect the most recent data available. Secondary outcomes included the frequency of primary care, cardiology, emergency department, and hospitalization encounters stratified by in-person versus virtual visits.

### Patient Characteristics of Interest

There is a large number of patient characteristics and equity covariates to consider; key features were selected by the study team who represent expertise in advanced heart failure, geriatrics, primary care, health equity, digital divide, clinical pharmacy, and hospital medicine. Features were selected based on univariate statistical significance, availability, completeness, and reliability of data collected in structured data fields, as well as if they were recognized as characteristics that influence how patients with HFrEF receive care.

Age and other patient characteristics were assessed at the start of the evaluation period. The National Center for Health Statistics (NCHS) Urban/Rural County designation scheme [11] was used to assign urban/rural status for each patient. Patients who were missing NCHS data but had a valid zip code within the state of Colorado were assigned urban/rural status based on their Colorado county of residence. Patients missing both NCHS and zip code data, or missing NCHS and living outside of Colorado, were assigned an urban/rural status of unknown. Due to large amounts of missing social determinants of health (SDoH) data (eg, transportation, food insecurity, homelessness), the Social Vulnerability Index (SVI) decimal value [12], based on zip code, was used to evaluate patient-level SDoH. The SVI is a widely recognized tool for measuring community-level vulnerabilities to environmental hazards. It integrates multiple socioeconomic and demographic variables to quantify social vulnerability. Determinants of the digital divide included data routinely collected within the EHR that have previously been found to be associated with the digital divide and were as follows: patient portal status, cell phone on file, and email on file in the EHR.

### Statistical Methods

Descriptive statistics were used to evaluate baseline patient demographics and clinical use data and outcomes. Categorical data were presented using counts and percentages, while discrete and continuous data points were presented using means, SDs, medians, minimums, maximums, and ranges where appropriate. Univariate analyses were performed both with virtual visits as a variable (received or did not receive) using the chi-square test for association and as a discrete outcome using the Wilcoxon rank-sum test to capture potentially important predictor variables that could influence the use or frequency of using virtual visits. Univariate comparisons for SVI, in-person primary care provider (PCP)/cardiology visits, and the most recent EF used simple logistic regression to model individual odds ratios (ORs).

For the primary outcome, a multivariable logistic regression model was used to model the odds of at least one virtual visit with a primary care or cardiology clinician during the study follow-up period. Covariates for this model were first chosen through clinical reasoning. Additional covariates exhibiting univariate significance were subsequently added to the adjusted model to evaluate their individual impact, as well as the impact on clinical covariates previously included. This multivariable model was also assessed for correlation between parameter estimates of predictor variables using a correlation matrix. Secondarily, in exploratory analyses, a negative binomial regression model was used to assess the incident rate of virtual visits during the evaluation period. The same patient characteristics of interest were adjusted for in this multivariable model as the adjusted logistic model mentioned above. These specific variables were estimated for their relationship with the rate of the outcome. Multiple variables were presented for evaluation of portal use (active patient portal status and portal ever registered), as well as a phone number listed on the patient file (home phone only, cell phone). Due to multicollinearity and incomplete data, portal registration and home phone only were excluded from all analyses. Patients with missing data for covariates were excluded from all multivariable analyses. Additional cross-tabulations and frequencies are presented in Multimedia Appendix 1 showing digital divide covariates stratified by demographic variables of interest. All statistical analyses were performed using SAS software (SAS Institute Inc).

## Results

### Overview

A total of 8481 patients were included in the analysis. This cohort reflects a largely urban population, where 7132 (84.1%) patients were categorized as residing in an urban location type based on zip code. Mean age was 65.90 years (SD 15.1) with 2400 (28.3%) participants older than 75 years. Included were 2807 (33.1%) female patients, 6608 (77.9%) White patients, 7356 (86.7%) non-Hispanic patients, 4711 (55.6%) patients on Medicare, and 3161 (37.3%) patients who had a most recent EF of ≤40%. Most patients (8035/8481, 94.7%) preferred English, and 396 (4.7%) of patients had ever needed or used an interpreter.

Variables that had a high percentage of missingness included employment status with 97.2% missing, and SDoH data including transportation, food insecurity, living conditions, homelessness, and housing status where <1% of patients had reliably reported data. Table 1 contains a description of the population at baseline.

**Table 1.** Baseline patient characteristics.

Characteristic total (N=8481)	Values	Missing or unknown
Age (years), mean (SD)	65.9 (15.1)	—^a^
Age (years), n (%)	0 (0)
>75	2400 (28.3)	
>85	653 (7.7)	
Sex, n (%)		2 (0.02)
Male	5672 (66.9)	
Female	2807 (33.1)	
Race, n (%)		12 (0.1)
White	6608 (77.9)	
Black	726 (8.6)	
Asian	135 (1.6)	
Other race	1000 (11.8)	
Ethnicity, n (%)		144 (1.7)
Hispanic	981 (11.6)	
Non-Hispanic	7356 (86.7)	
Preferred language, n (%)		
English	8035 (94.7)	—
Spanish	290 (3.4)	—
Other	156 (1.8)	—
Interpreter^b^, n (%)	396 (4.7)	—
Location type, n (%)		364 (4.3)
Urban	7132 (84.1)	
Rural	985 (11.6)	
Employment status, n (%)		8247 (97.2)
Employed	82 (1)	
Unemployed	152 (1.8)	
SSN^c^ on file, n (%)	7874 (92.8)	
Social Vulnerability Index, n (%)	0.4 (0.2)	643 (7.6)
Insurer/payor type, n (%)		5 (0.1)
Commercial	69 (0.8)	
Medicare	4711 (55.6)	
Medicaid	1,011 (11.9)	
Tricare	66 (0.8)	
Self-paying	1098 (13)	
Other	1521 (17.9)	
Digital divide indicators, n (%)		
Patient cell phone on file	7419 (87.5)	—
Active patient portal status	5930 (69.9)	—
Patient email on file	7522 (88.7)	—
Most recent ejection fraction, n (%)		
≤40	3161 (37.3)	—
≤30	1245 (14.7)	—
≤20	362 (4.3)	—
≤15	145 (1.7)	—
SDoH^d^ Data, n (%)		>8399 (99)
Transportation	9 (0.1)	
Food insecurity	7 (0.1)	
Living conditions	10 (0.1)	
Homeless	59 (0.7)	
Housing status	69 (0.8)	

a Not applicable.

b Interpreter means having ever needed or used an interpreter.

c SSN: social security number.

d SDOH: social determinants of health.

The large majority of patients had no cardiology (7938/8481, 93.6%) or primary care (7955/8481, 93.8%) virtual visits (Table 2). Of those that did receive at least one virtual visit, the mean number of visits was 1.4 (range 1‐10) for cardiology and 1.6 (range 1‐12) for primary care. In contrast, 2001 (23.6%) and 5957 (70.2%) of patients had no in-person visits in cardiology and primary care, respectively. Of those that did receive at least one in-person visit, the mean number of in-person visits was 2.8 (range 1‐38) for cardiology and 3.0 (range 1‐22) for primary care. We also explored how patients receive care over a combination of different visit types for the cohort as a whole and across multiple subgroups of patient characteristics to assess for differences in health care use. Table S1 in Multimedia Appendix 1 displays these data.

**Table 2.** Frequency of outpatient visit type by specialty (primary care or cardiology)*.*

Visit type	Virtual visit frequency				In-person visit frequency			
	Visit frequency per patients with ≥1 visit, mean (range)	Visit frequency per patients with ≥1 visit, median (IQR)	Patients with ≥1 virtual visit, n (%)	No visits (# pts with no virtual visits), n (%)	Visit frequency per patient with ≥1 visit, mean (range)	Visit frequency per patient with ≥1 visit, median (IQR)	Patients with ≥1 in-person visit, n (%)	No visits (# pts with no in-person visits), n (%)
Primary care	1.6 (1-12)	1 (1-2)	526 (6.2)	7955 (93.8)	3.0 (1-22)	2 (1-4)	2524 (29.8)	5957 (70.2)
Outpatient cardiology	1.4 (1-10)	1 (1-1)	543 (6.4)	7938 (93.6)	2.8 (1-38)	2 (1-3)	6480 (76.4)	2001 (23.6)

Table 3 lists the patient characteristics that were evaluated in the final model. In univariate comparisons (Table 3), the only significant findings were variables related to digital literacy, patient cell phone in EHR (OR 2.85, 95% CI 2.1-3.8; *P*<.001), patient email address in EHR (OR 9.29, 95% CI 5.6-15.5; *P*<.001), active patient portal status (OR 2.80, 95% CI 2.3-3.4*; P*<.001) and patients who had in-person primary care or cardiology visits had a higher mean number of virtual visits (6.32, SD 5.90) compared with those who did not (3.3, SD 3.82) (*P*<.001).

The mean number of virtual visits was 0.24 (SD 0.87) for those who had a cell phone listed in the EHR, compared with a mean of 0.07 (SD 0.40) virtual visits for those who did not (*P*<.001). The mean number of virtual visits was 0.24 (SD 0.90) for those who had an email address listed, compared with a mean of 0.02 (SD 0.15) virtual visits for those who did not (*P*<.001). The mean number of virtual visits was 0.11 (SD 0.58) for those who had an inactive patient portal status compared with a mean of 0.27 (SD 0.91) virtual visits for those who had an active patient portal status (*P*<.001).

**Table 3.** Unadjusted factors associated with receiving virtual care

Patient characteristic	Univariate categorical comparisons, OR^a^ (95% CI); *P* value	Univariate discrete comparisons (number of virtual PCP^b^ or Cardiology visits)	
		Mean (SD)	*P* value
Age (>75 years)^c^	1.0 (0.9-1.2); .6	No: 0.2 (0.8) Yes: 0.2 (0.9)	.6
Sex	1.1 (0.9-1.3); .2	Female: 0.2 (0.7) Male: 0.2 (0.9)	.2
Race	1.0 (0.9-1.2); .8	Non-White: 0.2 (0.7) White: 0.2 (0.9)	.8
Ethnicity	1.1 (0.9-1.4); .4	Hispanic: 0.2 (0.8) Non-Hispanic: 0.2 (0.8)	.5
Interpreter needed or used^c^	0.8 (0.6-1.1); .2	No: 0.2 (0.8) Yes: 0.2 (0.6)	.3
SSN^c^^,d^ on file	0.8 (0.6-1.1); .2	Yes: 0.2 (0.8) No: 0.2 (0.8)	.2
Insurance status	Medicaid versus Commercial/Medicare: 1.2 (1.0‐1.5); .1 Other versus Commercial/Medicare: 1.0 (0.8‐1.2); .8 Self-Pay versus Commercial/Medicare: 1.0 (0.8‐1.2); .9	Commercial/Medicare: 0.2 (0.8) Medicaid: 0.3 (0.9) Other: 0.2 (0.6) Self pay: 0.2 (0.9)	.3
Location type^e^	Unknown versus Rural: 1.0 (0.7‐1.5); >.99 Urban versus Rural: 1.1 (0.9‐1.3); .7	Rural: 0.2 (0.8) Unknown: 0.2 (0.7) Urban: 0.2 (0.8)	.9
Social Vulnerability Index^f^	1.0 (0.7-1.3); .8	Yes: 0.4 (0.2) No: 0.4 (0.2)	.8
Ejection fraction^f^	1.0 (1.0-1.0); .5	Yes: 46.1 (14) No: 46.3 (13.8)	.6
In-person PCP/cardiology visits^f^^,g^	1.1 (1.1-1.2); <.001	Yes: 6.3 (5.9) No: 3.3 (3.8)	≥.001
Death during study period^c^	1.0 (0.7-1.2); .07	No: 0.2 (0.8) Yes: 0.2 (0.8)	.8
Digital divide indicators			
Patient cell phone in EHR^c^	2.9 (2.1-3.8); <.001	No: 0.1 (0.4) Yes: 0.2 (0.9)	≤.001
Patient portal status	2.8 (2.3-3.4); <.001	Activated: 0.3 (0.9) Inactive: 0.1 (0.6)	≤.001
Patient email address in EHR^c^^,h^	9.3 (5.6-15.5); <.001	No: 0.02 (0.2) Yes: 0.2 (0.9)	≤.001

a OR: odds ratio.

b PCP: primary care provider.

c Yes/No response type.

d SSN: social security number.

e Location type was determined based on patient zip code.

f Odds ratios result from simple logistic regression.

g In-person PCP/cards visits refer to any in-person PCP/cardsvisits.

h EHR: electronic health record.

### Adjusted Model Results

Of the 8481 patients included in the study, 7837 (92.41%) had complete data with no missing covariate information. After adjusting for other covariates in the model, the primary multivariable logistic model showed significance in at least one virtual visit for a patient having a cell phone listed in the chart, email address on file, active patient portal status, and in-person visits with a primary care or cardiology clinician. Patients with a cell phone listed were at 68% higher odds of the outcome compared with patients without a cell phone listed (OR 1.68, 95% CI 1.23‐2.29). Patients with an active patient portal status were at 62% higher odds than their counterparts with an inactive or other patient portal status (OR 1.63, 95% CI 1.32‐2.00). Patients with an email address on file were at the highest odds of the outcome, with their odds being 5.18 times greater than patients without an email address on file (OR 5.18, 95% CI 2.96‐9.05). Last, for each in-person visit a patient had with a primary care or cardiology clinician, their odds of having the outcome of a virtual visit increased by 12.5% (OR 1.13, 95% CI 1.11‐1.14). Assessment of the correlation matrix did not exhibit high rates of correlation between parameter estimates of covariates in the model, with the highest correlation being 25% between active patient portal status and an email address on file.

Similar results were found when modeling the incident rate of virtual visits. The incident rate of a virtual visit was 7.36 times greater for patients with an email address on file compared with patients without an email address on file (95% CI 4.21‐12.87). Additionally, the incident rate was 60% higher for patients with an active patient portal status (95% CI 1.30‐1.97), and 87% higher for patients with a cell phone on file (95% CI 1.36‐2.57) compared with patients without an active patient portal status, and without a cell phone in file, respectively. Last, each in-person visit with a primary care or cardiology clinician was associated with a 14.4% increase in the rate of the outcome of a virtual visit (95% CI 1.12‐1.67). No other patient characteristics were significantly associated with the outcome of virtual visits. Table S2 in Multimedia Appendix 1 displays the parameter estimates for both the logistic and negative binomial models.

To further explore differences in access to digital tools, we cross-tabulated digital divide indicators including cell phone in the EHR, Portal Status, and email in the EHR by patient characteristics and did not see evidence of differences between the age, sex, race, ethnicity, rurality, use of an interpreter, insurance status, EF, or SVI (Table S3 in Multimedia Appendix 1).

## Discussion

### Principal Findings

In this post-COVID-19 pandemic cohort of patients with HFrEF, only a small number of patients with HFrEF had virtual visits with most outpatient visits occurring in person and more often in cardiology clinics than primary care. We also found that indicators of digital access such as having an email address on file or having an active EHR patient portal status were associated with having virtual visits. Given the relatively small number of minoritized patients within this cohort and the significant missingness of SDoH data collected, we were unable to fully evaluate for differences in patients receiving virtual care based on demographic and SDoH factors.

To our knowledge, this study is one of the first to offer estimates of virtual visits in patients with heart failure postpandemic and demonstrates a much lower use of virtual visits than during the pandemic [13]. Literature describing virtual visits for heart failure documents that while they were considered necessary during the pandemic [10] and can alleviate some burden of frequent visits, they are felt to have limitations in the care of heart failure particularly with regard to volume status assessment [14], which aligns with our findings of few virtual visits.

Although there is some evidence that virtual interventions can improve heart failure management [15], there is less evidence specifically on the impact of virtual synchronous visits alone for heart failure. For instance, telehealth interventions that incorporate multiple physiological measures tend to be more efficacious than interventions that rely on video visits alone [3]. In the case of heart failure management, it generally requires multiple physiologic measures to make informed decisions; thus, standard virtual visits without such telehealth interventions may not be sufficient. While there is currently limited evidence supporting the use of virtual care as a replacement for in-person care for heart failure management, the potential for virtual care augmented with telehealth interventions to improve outcomes and quality of care remains compelling [16].

Our finding that HFrEF patients are seen more often by cardiology than primary care is to our knowledge a novel contribution to the US heart failure literature. While estimates can be found in Swedish cohorts with 44% of patients managed by non-cardiologists [17], to our knowledge, no recent estimates have been published in US populations. That said, it seems likely that the frequency of follow-up with cardiology relative to primary care may vary substantially between health systems that serve communities with different payer mixes and access to specialists. Thus, these findings may have limited generalizability and should be evaluated in other health systems.

Another important finding was the large amount of missingness of the SDoH data including food insecurity, transportation, education, homelessness, and employment. This is a notable finding as these measures are known to predict health outcomes in many patient populations [18]. Although important, it is not surprising as the incompleteness of collecting SDoH data within the EHR is a well-recognized problem [19-21]. Without these data, needs such as housing and food insecurity cannot be addressed at the point of care, and additionally, disparities in care delivery and outcomes cannot be accurately measured at the health system level. The American Heart Association’s scientific statement on SDoH in patients with heart failure emphasizes the need to better measure SDoH through the integration of data capture within clinical practice [22]. It also emphasizes the importance of clinician education on the impact of SDoH on health outcomes to motivate the capture of these data during clinical care. However, additional strategies will likely be needed to obtain these data given the cognitive overload currently experienced by clinicians [23,24]. Natural language processing interventions that reduce the burden on clinicians and enhance patient engagement are being explored as a promising means of automating SDoH data capture [25]. More sophisticated and granular geospatial indices in addition to the overall social deprivation index are also becoming available [26]. In addition, recent changes to Medicare reimbursement, including payment for Patient Navigation and Community Health Improvement services, should also support expanded collection of SDoH data in the Medicare population [27].

Multiple studies have documented decreased access to virtual health interventions, or how the digital divide often disproportionally impacts racial and ethnic minorities and those of lower socioeconomic status [4]. This trend has been found in the heart failure population as well, although it is not as robustly documented [28,29]. However, this aspect of some telehealth interventions may be changing. A recently published analysis of the CONNECT-HF mHealth study found that younger, non-White, urban participants and those with either Medicaid or no insurance had higher mHealth access. This may be in part due to the technology used being cell phones which have been widely available for some time [28]. This is to some extent aligned with our own findings that virtual care was correlated with the use of cell phones. However, there are multiple possible explanations for this finding, and it should be interpreted with caution. It may be specific to our health system, the management of the disease of HFrEF, or reflect the dissemination of digital technology in society over time, among other possible explanations. Future studies should work to understand the replicability of this finding and identify the reasons for the results observed so the learning can be applied to other contexts in which the disparities still exist and inform interventions to resolve them.

### Strengths and Limitations

The study benefits from a large sample size, including academic, rural, suburban, and community settings. However, findings may not be fully generalizable to other settings that have different care delivery models, access to specialists, or payer mix. For instance, the majority of patients in this cohort had commercial insurance or Medicare, but this may be a reflection of these patients being older, which is generalizable for the HFrEF population. It is unclear to what extent our findings, such as high rates of ambulatory specialty care over primary care, would be found in a health system with a higher rate of patients without insurance or with Medicaid. As is the case with many US-based health systems, our findings are also limited to visits within the UCHealth health system and do not capture care received outside of the health system. Future studies should consider using alternate data sources such as claims data or health information exchanges to capture care received across more than one health system. Additionally, we used EF alone and not diagnosis codes to define our cohort and thus may have included some patients with very transient decreases in EF due to other reasons (eg myocarditis). However, this likely only represents a small number of patients.

Missing SDoH data is both an important finding and a limitation of this study. It has been mitigated as a limitation by the use of the SVI within our analysis. However, our inability to accurately measure these patient characteristics restricts our ability to fully evaluate for equity in care delivery. Notably, this data set’s limitations impact all evaluations of care within this large health system and likely reflect the current inadequacies of SDoH data capture in many other health systems [30]. Additionally, the low number of specific racial and ethnic minorities within the study cohort that participated in virtual visits limits our ability to detect differences in virtual visit use in these patient groups.

### Conclusions

In conclusion, patients with HFrEF were more likely to receive care in person and with cardiology than virtually or with primary care. While patients with higher levels of digital access were more likely to be seen virtually, indicators of the digital divide did not correlate with race, ethnicity, or insurance status. Future studies should explore the replicability of these findings.

## Supplementary material

10.2196/70414 Multimedia Appendix 1 Visit type by patient demographics and regression model parameter estimates.

## Abbreviations

EF: ejection fraction
EHR: electronic health record
HFrEF: heart failure with reduced ejection fraction
NCHS: National Center for Health Statistics
OR: odds ratio
PCP: primary care provider
SDoH: social determinants of health
SVI: Social Vulnerability Index
