# Supplementary material for: Frequency and Predictors of Virtual Visits in Patients With Heart Failure Within a Large Health System: Retrospective Cohort Study
Source: J Med Internet Res. 2025 Aug 12;27:e70414. doi: 10.2196/70414 (PMC12501900; doi:10.2196/70414)
Supplement: Multimedia Appendix 1 [file jmir-v27-e70414-s001.docx]

| **Table S1.***.* Visit Type by Patient Characteristic. | | | | | | | | | | | | | | | | | | | | | | | | |
| --- | --- | --- | --- | --- | --- | --- | --- | --- | --- | --- | --- | --- | --- | --- | --- | --- | --- | --- | --- | --- | --- | --- | --- | --- |
|  | |  | **No Visits** | | **IP/ED visit, no OP*** | | **IP/ED with OP*** | | **IP/ED visit, PCP visit, No Cards** | | **IP /ED visit, Cards visit, no PCP** | | **OP only*** | | **PCP only** | | **Cards only** | | **Virtual OP only*** | | **Virtual PCP only** | | **Virtual Cards only** | |
| **Variable** | **Response** | **Total N** | **N** | **%** | **N** | **%** | **N** | **%** | **N** | **%** | **N** | **%** | **N** | **%** | **N** | **%** | **N** | **%** | **N** | **%** | **N** | **%** | **N** | **%** |
| Total |  | *8481* | *1316* | *15.5%* | *337* | *4%* | *2981* | *35.25%* | *113* | *1.3%* | *399* | *4.9%* | *3847* | *45.4%* | *146* | *1.7%* | *2573* | *30.3%* | *55* | *0.7%* | *6* | *0.1%* | *44* | *0.5%* |
| Age | Mean(SD) |  | 66.3 | 15.3 | 66.8 | 14.7 | 65.7 | 15.2 | 67.9 | 13.8 | 66.3 | 15.5 | 65.9 | 15.0 | 64.5 | 16.9 | 66.0 | 15.0 | 66.0 | 13.0 | 69.8 | 15.3 | 66.2 | 13.0 |
| Ethnicity | Hispanic | 981 | 151 | 15.4% | 38 | 3.9% | 379 | 38.6% | 13 | 1.3% | 53 | 5.4% | 413 | 42.1% | 20 | 2.0% | 260 | 26.5% | 4 | 0.4% | . | . | 4 | 0.4% |
|  | Non-Hispanic | 7356 | 1147 | 15.6% | 294 | 4.0% | 2542 | 34.6% | 97 | 1.3% | 339 | 4.6% | 3373 | 45.9% | 124 | 1.7% | 2273 | 30.9% | 50 | 0.7% | 5 | 0.1% | 40 | 0.5% |
|  | Unknown/ other | 144 | 18 | 12.5% | 5 | 3.5% | 60 | 41.7% | 3 | 2.1% | 7 | 4.9% | 61 | 42.4% | 2 | 1.4% | 40 | 27.8% | 1 | 0.7% | 1 | 0.7% | . | . |
| Insurance | Commercial | 69 | 9 | 13.0% | 3 | 4.4% | 22 | 31.9% | . | . | 2 | 2.9% | 35 | 50.7% | . | . | 24 | 34.8% | . | . | . | . | . | . |
|  | Medicaid | 1011 | 158 | 15.6% | 37 | 3.7% | 372 | 36.8% | 15 | 1.5% | 42 | 4.2% | 444 | 43.9% | 24 | 2.4% | 293 | 29% | 9 | 0.9% | 1 | 0.1% | 6 | 0.6% |
|  | Medicare | 4711 | 729 | 15.5% | 199 | 4.2% | 1646 | 34.9% | 72 | 1.5% | 227 | 4.8% | 2137 | 45.4% | 75 | 1.6% | 1445 | 30.7% | 30 | 0.6% | 3 | 0.1% | 27 | 0.6% |
|  | Other | 1521 | 235 | 15.5% | 52 | 3.4% | 508 | 33.4% | 16 | 1.1% | 71 | 4.7% | 726 | 47.7% | 30 | 2.0% | 472 | 31% | 7 | 0.5% | . | . | 6 | 0.4% |
|  | Self Pay | 1098 | 178 | 16.2% | 44 | 4.0% | 398 | 36.3% | 9 | 0.8% | 54 | 4.9% | 478 | 43.5% | 14 | 1.3% | 325 | 29.6% | 9 | 0.8% | 2 | 0.2% | 5 | 0.5% |
|  | Tricare | 66 | 5 | 7.6% | 2 | 3.0% | 34 | 51.5% | 1 | 1.5% | 3 | 4.6% | 25 | 37.9% | 3 | 4.6% | 12 | 18.2% | . | . | . | . | . | . |
|  | Unknown | 5 | 2 | 40.0% | . | . | 1 | 20% | . | . | . | . | 2 | 40% | . | . | 2 | 40% | . | . | . | . | . | . |
| Language | English | 8035 | 1239 | 15.4% | 319 | 4.0% | 2844 | 35.4% | 109 | 1.4% | 383 | 4.8% | 3633 | 45.2% | 137 | 1.7% | 2440 | 30.4% | 52 | 0.6% | 5 | 0.1% | 43 | 0.5% |
|  | Other | 156 | 27 | 17.3% | 6 | 3.9% | 48 | 30.8% | 2 | 1.3% | 4 | 2.6% | 75 | 48.1% | 2 | 1.3% | 49 | 31.4% | 2 | 1.3% | 1 | 0.6% | . | . |
|  | Spanish | 290 | 50 | 17.2% | 12 | 4.1% | 89 | 30.7% | 2 | 0.7% | 12 | 4.1% | 139 | 47.9% | 7 | 2.4% | 84 | 29% | 1 | 0.3% | . | . | 1 | 0.3% |
| Race | Asian | 135 | 26 | 19.3% | 7 | 5.2% | 49 | 36.3% | . | . | 7 | 5.2% | 53 | 39.3% | 1 | 0.7% | 36 | 26.7% | 1 | 0.7% | . | . | . | . |
|  | Black | 726 | 108 | 14.9% | 30 | 4.1% | 246 | 33.9% | 7 | 1% | 33 | 4.6% | 342 | 47.1% | 15 | 2.1% | 225 | 31% | 6 | 0.8% | . | . | 6 | 0.8% |
|  | Missing | 12 | 3 | 25.0% | . | . | 4 | 33.3% | 1 | 8.3% | . | . | 5 | 41.7% | . | . | 4 | 33.3% | . | . | . | . | . | . |
|  | Other | 1000 | 155 | 15.5% | 39 | 3.9% | 362 | 36.2% | 14 | 1.4% | 54 | 5.4% | 444 | 44.4% | 20 | 2.0% | 289 | 28.9% | 5 | 0.5% | . | . | 5 | 0.5% |
|  | White | 6608 | 1024 | 15.5% | 261 | 4.0 % | 2320 | 35.1% | 91 | 1.4% | 305 | 4.6% | 3003 | 45.4% | 110 | 1.7% | 2019 | 30.6% | 43 | 0.6% | 6 | 0.1% | 33 | 0.5% |
| Sex | Female | 2807 | 446 | 15.9% | 111 | 4.0% | 993 | 35.4% | 39 | 1.4% | 137 | 4.9% | 1257 | 44.8% | 59 | 2.1% | 806 | 28.7% | 20 | 0.7% | 1 | 0.0% | 17 | 0.6% |
|  | Male | 5672 | 870 | 15.3% | 226 | 4.0% | 1988 | 35.1% | 74 | 1.3% | 262 | 4.6% | 2588 | 45.6% | 87 | 1.5% | 1765 | 31.1% | 35 | 0.6% | 5 | 0.1% | 27 | 0.5% |
|  | Unknown | 2 | . | . | . | . | . | . | . | . | . | . | 2 | 100 % | . | . | 2 | 100% | . | . | . | . | . | . |
| Rurality | Rural | 985 | 199 | 20.2% | 21 | 2.1% | 237 | 24.1% | 9 | 0.9% | 45 | 4.6% | 528 | 53.6% | 6 | 0.6% | 455 | 46.2% | 10 | 1% | . | . | 9 | 0.9% |
|  | Unknown | 364 | 61 | 16.8% | 15 | 4.1% | 132 | 36.3% | 6 | 1.7% | 22 | 6 % | 156 | 42.9% | 6 | 1.7% | 113 | 31 % | . | . | . | . | . | . |
|  | Urban | 7132 | 1056 | 14.8% | 301 | 4.2% | 2612 | 36.6% | 98 | 1.4% | 332 | 4.7% | 3163 | 44.4% | 134 | 1.9% | 2005 | 28.1% | 45 | 0.6% | 6 | 0.1% | 35 | 0.4% |
| IP=Inpatient, ED=Emergency Department, OP = Outpatient, PCP=Primary care provider, Cards=Cardiology  “.” = N=0  *OP refers to any outpatient cardiology or outpatient primary care visit, in-person or virtual. | | | | | | | | | | | | | | | | | | | | | | | | |

| **Table S2.** Parameter Estimates and Confidence Intervals for Variables in Logistic and Negative Binomial Regression Models. | | | | | | | | |
| --- | --- | --- | --- | --- | --- | --- | --- | --- |
| **Model Type** | **Logistic Modeling*** | | | | **Negative Binomial Modeling**** | | | |
| Effect | Estimate | 95% Confidence Limits | | P-value | Estimate | 95% Confidence Limits | | p-values |
| Age > 75  No vs Yes | 0.9 | 0.8 | 1.1 | 0.3 | 1.0 | 0.8 | 1.2 | 0.7 |
| Sex  Male vs Female | 1.1 | 1.0 | 1.3 | 0.2 | 1.2 | 1.0 | 1.4 | 0.1 |
| Urban/Rural  Unknown vs Rural | 0.9 | 0.6 | 1.3 | 0.5 | 0.9 | 0.6 | 1.5 | 0.7 |
| Urban/Rural  Urban   vs Rural | 0.8 | 0.7 | 1.1 | 0.1 | 0.9 | 0.7 | 1.1 | 0.3 |
| SVI (per 1 unit increase) | 1.0 | 0.7 | 1.3 | 0.8 | 0.8 | 0.6 | 1.2 | 0.4 |
| SSN on File  No vs Yes | 0.8 | 0.6 | 1.0 | 0.1 | 0.8 | 0.6 | 1.1 | 0.2 |
| Race  Non-White vs White | 1.0 | 0.9 | 1.3 | 0.8 | 0.9 | 0.8 | 1.0 | 0.2 |
| Ethnicity  Hispanic vs Non-Hispanic or Other | 0.9 | 0.7 | 1.2 | 0.5 | 1.0 | 0.9 | 1.2 | 0.9 |
| Insurance  Medicaid vs Commercial/Medicare | 1.3 | 1.0 | 1.6 | 0.04 | 1.3 | 1.0 | 1.7 | 0.02 |
| Insurance  Other  vs Commercial/Medicare | 1.0 | 0.8 | 1.3 | 0.9 | 0.9 | 0.7 | 1.2 | 0.5 |
| Insurance  Self Pay vs Commercial/Medicare | 1.0 | 0.8 | 1.3 | 0.7 | 1.2 | 1.0 | 1.6 | 0.1 |
| Interpreter  No vs Yes | 1.1 | 0.7 | 1.6 | 0.7 | 1.0 | 0.9 | 1.3 | 0.7 |
| Patient Cell Phone In Chart  Yes vs No | 1.7 | 1.2 | 2.3 | 0.001 | 1.9 | 1.4 | 2.6 | .0001 |
| Portal status  Activated vs Inactive | 1.6 | 1.3 | 2.0 | <.0001 | 1.6 | 1.3 | 2.0 | <.0001 |
| Patient Email Address on File  Yes vs No | 5.2 | 3.0 | 9.1 | <.0001 | 7.4 | 4.2 | 12.9 | <.0001 |
| Death  0 (No) vs 1 (Yes) | 1.0 | 0.7 | 1.3 | 0.9 | 1.0 | 0.8 | 1.1 | 0.8 |
| Ejection Fraction  (Most Recent) | 1.0 | 1.0 | 1.0 | 1.0 | 1.0 | 1.0 | 1.0 | 0.1 |
| In Person PCP/Cards Visits (per 1 in person visit) | 1.1 | 1.1 | 1.1 | <.0001 | 1.1 | 1.1 | 1.2 | <.0001 |

*Interpretation: Binary logistic regression was used to model the odds of having a virtual visit with a primary care or cardiology related provider during the study follow-up period. This multivariable model included all variables listed above, chosen through clinical reasoning and/or univariate statistical significance. Home phone only, any phone listed in chart, and active MyChart use were excluded from this model due to collinearity with other variables in the model. After adjusting for other covariates in the model, a cell phone listed in chart, email address on file, MyChart status, and in person visits with a primary care or cardiology related provider were found to be significantly associated with the outcome (See Type 3 Effects Above). Patients with a cell phone listed were at 68% higher odds of the outcome compared to patients without a cell phone listed (OR: 1.68 95% CI: 1.23 – 2.29). Patients with an active MyChart were at 62% higher odds than their counterparts with and inactive or other MyChart (1.63, 1.32 – 2.00). Patients with an email address on file were at the highest odds of the outcome, with their odds being 5.18 times greater than patients without an email address on file (5.18, 2.96 – 9.05). Lastly, for each in person visit a patient had with a primary care or cardiology related provider, their odds of having the outcome of a virtual visit with a primary care or cardiology related provider increased by 12.5% (1.13, 1.11 – 1.14).

** Interpretation: Negative binomial regression was used to model the discrete count of virtual visits with a primary care or cardiology provider during the study follow-up period. This multivariable model included all variables listed above, chosen through clinical reasoning and/or univariate statistical significance. After adjusting for other covariates in the model, a cell phone listed in chart, email address on file, MyChart status, and in person visits with a primary care or cardiology related provider were found to be significantly associated with the outcome (See Type 3 Effects Above). These specific variables were estimated for their estimated relationship with the rate of the outcome.

The incident rate of the virtual outcome was 7.36 times greater for patients with an email address on file compared to patients without an email address on file (95% CI: 4.21 – 12.87). Additinoally, the incident rate was 60% higher for patients with an active MyChart (1.30 – 1.97), and 87% higher for patients with a cell phone on file (1.36 – 2.57) compared to patients without and active MyChart, and without a cell phone in file, respectively. Lastly, each in person visit with a primary care or cardiology related provider was associated with a 14.4% increase in the rate of the outcome of a virtual visit with one of these providers (1.12 – 1.67).

| **Table S3.** Digital divide indicators by patient characteristic. | | | | | | | |
| --- | --- | --- | --- | --- | --- | --- | --- |
|  |  | **Cell Phone in EHR** | | **Portal Status** | | **Email Address in EHR** | |
|  |  | **No** | **Yes** | **Active** | **Inactive** | **No** | **Yes** |
| **Variable** | **Response** | **N (%)** | **N (%)** | **N (%)** | **N (%)** | **N (%)** | **N (%)** |
| Age > 75 | No | 770 (12.7%) | 5,311 (87.3%) | 4,246 (69.8%) | 1,835 (30.2%) | 674 (11.1%) | 5,407 (89.0%) |
|  | Yes | 292 (12.2%) | 2,108 (87.8%) | 1,684 (70.2%) | 716 (29.8%) | 285 (11.9%) | 2,115 (88.1%) |
| Sex | Female | 348 (12.4%) | 2,459 (87.6%) | 1,984 (70.7%) | 823 (29.3%) | 291 (10.4%) | 2,516 (89.7%) |
|  | Male | 714 (12.6%) | 4,958 (87.4%) | 3,945 (69.6%) | 1,727 (30.5%) | 668 (11.8%) | 5,004 (88.2%) |
|  | Unknown |  | 2 (100.0%) | 1 (50.00%) | 1 (50.00%) |  | 2 (100.0%) |
| Location Type | Rural | 167 (17.0%) | 818 (83.1%) | 589 (59.8%) | 396 (40.2%) | 186 (18.9%) | 799 (81.1%) |
|  | Unknown | 50 (13.7%) | 314 (86.3%) | 252 (69.2%) | 112 (30.8%) | 47 (12.9%) | 317 (87.1%) |
|  | Urban | 845 (11.9%) | 6,287 (88.2%) | 5,089 (71.4%) | 2,043 (28.7%) | 726 (10.2%) | 6,406 (89.9%) |
| SSN on file | No | 985 (12.5%) | 6,889 (87.5%) | 5,500 (69.9%) | 2,374 (30.2%) | 898 (11.4%) | 6,976 (88.6%) |
|  | Yes | 77 (12.7%) | 530 (87.3%) | 430 (70.8%) | 177 (29.2%) | 61 (10.1%) | 546 (90.0%) |
| Race | Non-White | 224 (12.0%) | 1,649 (88.0%) | 1,319 (70.4%) | 554 (29.6%) | 189 (10.1%) | 1,684 (89.9%) |
|  | White | 838 (12.7%) | 5,770 (87.3%) | 4,611 (69.8%) | 1,997 (30.2%) | 770 (11.7%) | 5,838 (88.4%) |
| Ethnicity | Hispanic | 123 (12.5%) | 858 (87.5%) | 679 (69.2%) | 302 (30.8%) | 109 (11.1%) | 872 (88.9%) |
|  | Non-Hispanic or Other | 939 (12.5%) | 6,561 (87.5%) | 5,251 (70.0%) | 2,249 (30.0%) | 850 (11.3%) | 6,650 (88.7%) |
| Interpreter | No | 1,008 (12.5%) | 7,077 (87.5%) | 5,656 (70.0%) | 2,429 (30.0%) | 918 (11.4%) | 7,167 (88.7%) |
|  | Yes | 54 (13.6%) | 342 (86.4%) | 274 (69.2%) | 122 (30.8%) | 41 (10.4%) | 355 (89.7%) |
| Insurance Status | Commercial/Medicare | 595 (12.5%) | 4,185 (87.6%) | 3,353 (70.2%) | 1,427 (29.9%) | 555 (11.6%) | 4,225 (88.4%) |
|  | Medicaid | 137 (13.6%) | 874 (86.5%) | 713 (70.5%) | 298 (29.5%) | 104 (10.3%) | 907 (89.7%) |
|  | Other | 209 (13.1%) | 1,383 (86.9%) | 1,096 (68.8%) | 496 (31.2%) | 169 (10.6%) | 1,423 (89.4%) |
|  | Self Pay | 121 (11.0%) | 977 (89.0%) | 768 (70.0%) | 330 (30.1%) | 131 (11.9%) | 967 (88.1%) |
| Ejection Fraction <= 40% | No | 631 (11.9%) | 4,689 (88.1%) | 3,870 (72.7%) | 1,450 (27.3%) | 545 (10.2%) | 4,775 (89.8%) |
|  | Yes | 431 (13.6%) | 2,730 (86.4%) | 2,060 (65.2%) | 1,101 (34.8%) | 414 (13.1%) | 2,747 (86.9%) |
| SVI | Mean(Range),  Median(IQR) | 0.4 (0.0 – 1.0), 0.4 (0.2 - 0.6) | 0.4 (0.0 – 1.0), 0.4, (0.2 - 0.6) | 0.4 (0.0 – 1.0), 0.4 (0.2 - 0.6) | 0.4 (0.0 – 1.0), 0.4 (0.2 - 0.6) | 0.4 (0.0 - 0.9), 0.4 (0.2 - 0.6) | 0.4 (0.0 – 1.0) 0.4 (0.2 - 0.60) |
